# 2-(1*H*-Tetra­zol-1-yl)acetic acid monohydrate

**DOI:** 10.1107/S1600536812033090

**Published:** 2012-07-28

**Authors:** Wen-Xiang Wang

**Affiliations:** a Ordered Matter Science Research Center, College of Chemistry and Chemical, Engineering, Southeast University, Nanjing 211189, People’s Republic of China

## Abstract

The crystal structure of the title compound, C_3_H_4_N_4_O_2_·H_2_O, exhibits O—H⋯O and O—H⋯N hydrogen bonds, which lead to the formation of a two-dimensional network parallel to the *bc* plane. The dihedral angle between the ring and the carboxylic acid group is 84.6 (14)°.

## Related literature

For the use of 2-(1*H*-tetra­zol-1-yl) acetic acid as a pharmaceutical inter­mediate, see: Gunnlaugsson & Stomeo (2007 ▶). For its coordination properties, see: Ghosh & Bharadwaj (2004 ▶). For the synthesis, see: Jústiz *et al.* (1997 ▶).
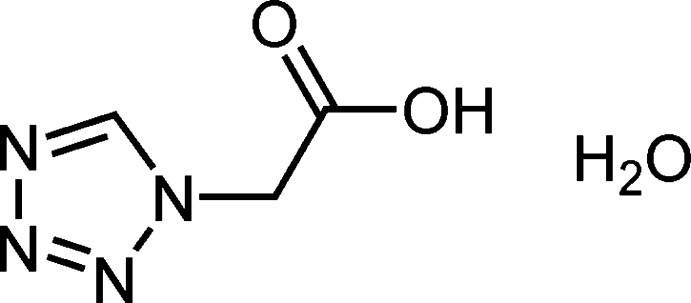


## Experimental

### 

#### Crystal data

C_3_H_4_N_4_O_2_·H_2_O
*M* *_r_* = 146.12
Orthorhombic, 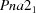

*a* = 12.618 (3) Å
*b* = 5.1871 (10) Å
*c* = 9.874 (2) Å
*V* = 646.2 (2) Å^3^
*Z* = 4
Mo *K*α radiation
μ = 0.13 mm^−1^
*T* = 293 K
0.26 × 0.23 × 0.19 mm

#### Data collection

Rigaku SCXmini diffractometer
Absorption correction: multi-scan (*CrystalClear*; Rigaku, 2005 ▶) *T*_min_ = 0.965, *T*_max_ = 0.983
6216 measured reflections
786 independent reflections
715 reflections with *I* > 2σ(*I*)
*R*_int_ = 0.031

#### Refinement

*R*[*F* ^2^ > 2σ(*F* ^2^)] = 0.030
*wR*(*F* ^2^) = 0.069
*S* = 1.17
786 reflections
103 parameters
1 restraint
H atoms treated by a mixture of independent and constrained refinement
Δρ_max_ = 0.11 e Å^−3^
Δρ_min_ = −0.12 e Å^−3^

### 

Data collection: *CrystalClear* (Rigaku, 2005 ▶); cell refinement: *CrystalClear*; data reduction: *CrystalClear*; program(s) used to solve structure: *SHELXS97* (Sheldrick, 2008 ▶); program(s) used to refine structure: *SHELXL97* (Sheldrick, 2008 ▶); molecular graphics: *DIAMOND* (Brandenburg & Putz, 2005 ▶); software used to prepare material for publication: *SHELXL97*.

## Supplementary Material

Crystal structure: contains datablock(s) I, global. DOI: 10.1107/S1600536812033090/fy2060sup1.cif

Structure factors: contains datablock(s) I. DOI: 10.1107/S1600536812033090/fy2060Isup2.hkl

Supplementary material file. DOI: 10.1107/S1600536812033090/fy2060Isup3.cml

Additional supplementary materials:  crystallographic information; 3D view; checkCIF report

## Figures and Tables

**Table 1:** Hydrogen-bond geometry (Å, °)

*D*—H⋯*A*	*D*—H	H⋯*A*	*D*⋯*A*	*D*—H⋯*A*
O3—H3*B*⋯N2	0.83 (4)	2.11 (4)	2.864 (3)	150 (3)
O1—H1*A*⋯O3^i^	0.89 (4)	1.72 (4)	2.603 (3)	175 (4)
O3—H3*A*⋯O2^ii^	0.76 (3)	2.00 (3)	2.752 (3)	168 (3)

Symmetry codes: (i) 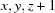; (ii) 
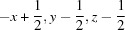
.

**Fig. 1.**
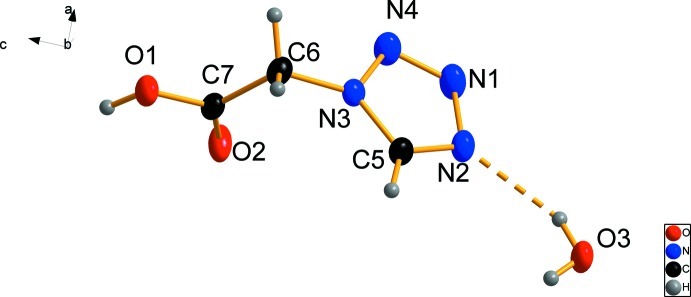
Asymmetric unit with anisotropic ellipsoid representation, shown with the atom labeling scheme. Ellispoids are shown at the 50% probability level.

**Fig. 2.**
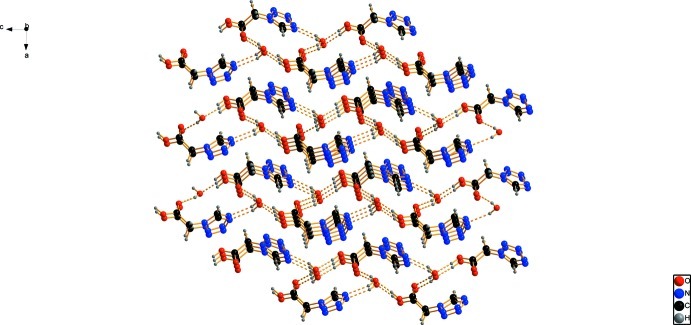
Packing diagram for the title compound. Hydrogen bonds are denoted by dashed lines.

## supplementary crystallographic information

### Comment

2-(1*H*-tetrazol-1-yl) acetic acid is an important raw material to prepare a variety of antibiotic drugs, and is widely used as an important pharmaceutical intermediate (Gunnlaugsson & Stomeo, 2007). In recent years, researchers found that tetrazole acetic acid has excellent coordination properties (Ghosh & Bharadwaj, 2004). We report here the crystal structure of the title compound (Fig. 1).

In the title compound the carboxyl functional group is almost perpendicular to the tetrazole heterocycle with a dihedral angle of 87.3 (2)°. The O3—H3B···N2 hydrogen bond anchors the water molecule to the tetrazole heterocycle. Two intermolecular hydrogen bonds (O1—H1A···O3 and O3—H3A···O2) connect the water molecule and two carboxyl groups from the neighboring asymmetric unit, forming layers with louver-like network (Fig. 2).

### Experimental

A procedure similar to the previously published method of Jústiz *et al.* (1997) was applied. To a solution of 2-aminoacetic acid (7.5 g, 0.1 mol) in 50 ml acetic acid was added triethoxymethane (32.4 g, 0.22 mol) and sodium azide (7.15 g, 0.11 mol). The mixture was refluxed for 3.0 h at 80°C. Active carbon was used to discolor the mixture, which was then refluxed for another 10 minutes. Heating was stopped and cooled to room temperature, the mixture was filtered to remove the active carbon and concentrated hydrochloric acid was trickled into the filtrate and a white solid product precipitated out. The precipitate was extracted by ethyl acetate and washed with saturated solution of sodium bicarbonate, brine, and then dried over MgSO_4_. Evaporation of the solvent in vacuum afforded the 2-(1*H*-tetrazol-1-yl) acetic acid compound. The pale yellow single crystals of the title compound suitable for X-ray analysis were obtained by slow evaporation of a solution in the 3:7 (v/v) mixture of petroleum ether and ethyl acetate.

### Refinement

In the absence of significant anomalous dispersion effects, Friedel pairs were averaged. Hydrogen atom positions were calculated geometrically and were set to ride on the associated C atoms, with *U*_iso_(H)= 1.2 *U*_iso_(C). The H atoms on O were located in difference electron density maps and were refined freely with isotropic displacement parameters.

### Crystal data

**Table:**

C_3_H_4_N_4_O_2_·H_2_O	*F*(000) = 304
*M**_r_* = 146.12	*D*_x_ = 1.502 Mg m^−^^3^
Orthorhombic, *P**n**a*2_1_	Mo *K*α radiation, λ = 0.71073 Å
Hall symbol: P 2c -2n	Cell parameters from 6216 reflections
*a* = 12.618 (3) Å	θ = 3.2–27.5°
*b* = 5.1871 (10) Å	µ = 0.13 mm^−^^1^
*c* = 9.874 (2) Å	*T* = 293 K
*V* = 646.2 (2) Å^3^	Needle, pale yellow
*Z* = 4	0.26 × 0.23 × 0.19 mm

### Data collection

**Table:**

Rigaku SCXmini diffractometer	786 independent reflections
Radiation source: fine-focus sealed tube	715 reflections with *I* > 2σ(*I*)
Graphite monochromator	*R*_int_ = 0.031
ω scans	θ_max_ = 27.5°, θ_min_ = 3.2°
Absorption correction: multi-scan (*CrystalClear*; Rigaku, 2005)	*h* = −16→16
*T*_min_ = 0.965, *T*_max_ = 0.983	*k* = −6→6
6216 measured reflections	*l* = −12→12

### Refinement

**Table:**

Refinement on *F*^2^	Primary atom site location: structure-invariant direct methods
Least-squares matrix: full	Secondary atom site location: difference Fourier map
*R*[*F*^2^ > 2σ(*F*^2^)] = 0.030	Hydrogen site location: inferred from neighbouring sites
*wR*(*F*^2^) = 0.069	H atoms treated by a mixture of independent and constrained refinement
*S* = 1.17	*w* = 1/[σ^2^(*F*_o_^2^) + (0.029*P*)^2^ + 0.0792*P*] where *P* = (*F*_o_^2^ + 2*F*_c_^2^)/3
786 reflections	(Δ/σ)_max_ < 0.001
103 parameters	Δρ_max_ = 0.11 e Å^−^^3^
1 restraint	Δρ_min_ = −0.12 e Å^−^^3^

### Special details

**Table:**

Geometry. All e.s.d.'s (except the e.s.d. in the dihedral angle between two l.s. planes) are estimated using the full covariance matrix. The cell e.s.d.'s are taken into account individually in the estimation of e.s.d.'s in distances, angles and torsion angles; correlations between e.s.d.'s in cell parameters are only used when they are defined by crystal symmetry. An approximate (isotropic) treatment of cell e.s.d.'s is used for estimating e.s.d.'s involving l.s. planes.
Refinement. Refinement of *F*^2^ against ALL reflections. The weighted *R*-factor *wR* and goodness of fit *S* are based on *F*^2^, conventional *R*-factors *R* are based on *F*, with *F* set to zero for negative *F*^2^. The threshold expression of *F*^2^ > σ(*F*^2^) is used only for calculating *R*-factors(gt) *etc*. and is not relevant to the choice of reflections for refinement. *R*-factors based on *F*^2^ are statistically about twice as large as those based on *F*, and *R*- factors based on ALL data will be even larger.

### Fractional atomic coordinates and isotropic or equivalent isotropic displacement parameters (Å^2^)

**Table:**

	*x*	*y*	*z*	*U*_iso_*/*U*_eq_	
O1	0.38513 (14)	0.5364 (3)	1.01953 (17)	0.0450 (4)	
O2	0.30421 (14)	0.8326 (3)	0.89450 (17)	0.0525 (5)	
N2	0.37640 (16)	0.8301 (4)	0.4743 (2)	0.0436 (5)	
C5	0.36860 (19)	0.6540 (4)	0.5678 (2)	0.0381 (5)	
H5A	0.3281	0.5047	0.5614	0.046*	
N4	0.47431 (18)	0.9471 (4)	0.6448 (2)	0.0497 (5)	
C7	0.37025 (18)	0.6679 (4)	0.9087 (2)	0.0358 (5)	
N1	0.44272 (18)	1.0105 (4)	0.5252 (2)	0.0527 (6)	
N3	0.42748 (14)	0.7205 (3)	0.67377 (18)	0.0334 (4)	
C6	0.44751 (19)	0.5888 (4)	0.8003 (2)	0.0379 (5)	
H6A	0.5190	0.6270	0.8302	0.046*	
H6B	0.4424	0.4042	0.7860	0.046*	
O3	0.27445 (17)	0.7053 (4)	0.22354 (17)	0.0509 (5)	
H1A	0.344 (3)	0.595 (6)	1.086 (4)	0.079 (11)*	
H3A	0.246 (2)	0.600 (6)	0.263 (4)	0.062 (10)*	
H3B	0.310 (3)	0.789 (6)	0.279 (4)	0.062 (10)*	

### Atomic displacement parameters (Å^2^)

**Table:**

	*U*^11^	*U*^22^	*U*^33^	*U*^12^	*U*^13^	*U*^23^
O1	0.0495 (9)	0.0525 (10)	0.0330 (9)	0.0046 (8)	−0.0002 (8)	0.0146 (8)
O2	0.0605 (10)	0.0645 (11)	0.0327 (9)	0.0259 (9)	0.0079 (8)	0.0131 (9)
N2	0.0554 (12)	0.0453 (11)	0.0303 (9)	−0.0005 (9)	0.0026 (9)	0.0036 (9)
C5	0.0438 (14)	0.0403 (12)	0.0303 (11)	−0.0033 (9)	0.0011 (9)	−0.0052 (9)
N4	0.0678 (14)	0.0421 (11)	0.0393 (11)	−0.0149 (10)	0.0003 (11)	−0.0023 (9)
C7	0.0409 (12)	0.0368 (11)	0.0298 (11)	−0.0010 (10)	−0.0039 (9)	0.0036 (10)
N1	0.0729 (14)	0.0454 (11)	0.0396 (12)	−0.0118 (10)	0.0050 (12)	0.0042 (10)
N3	0.0402 (9)	0.0325 (9)	0.0276 (8)	0.0000 (8)	0.0031 (8)	−0.0036 (7)
C6	0.0446 (12)	0.0366 (11)	0.0326 (11)	0.0070 (11)	−0.0046 (10)	−0.0011 (9)
O3	0.0612 (11)	0.0625 (12)	0.0289 (9)	−0.0116 (10)	−0.0018 (8)	0.0107 (9)

### Geometric parameters (Å, º)

**Table:**

O1—C7	1.303 (3)	N4—N3	1.346 (3)
O1—H1A	0.89 (4)	C7—C6	1.505 (3)
O2—C7	1.202 (3)	N3—C6	1.446 (3)
N2—C5	1.303 (3)	C6—H6A	0.9700
N2—N1	1.352 (3)	C6—H6B	0.9700
C5—N3	1.329 (3)	O3—H3A	0.76 (3)
C5—H5A	0.9300	O3—H3B	0.83 (4)
N4—N1	1.289 (3)		
			
C7—O1—H1A	111 (2)	C5—N3—N4	107.74 (19)
C5—N2—N1	105.6 (2)	C5—N3—C6	130.92 (19)
N2—C5—N3	109.49 (19)	N4—N3—C6	121.31 (19)
N2—C5—H5A	125.3	N3—C6—C7	111.87 (17)
N3—C5—H5A	125.3	N3—C6—H6A	109.2
N1—N4—N3	106.37 (19)	C7—C6—H6A	109.2
O2—C7—O1	124.8 (2)	N3—C6—H6B	109.2
O2—C7—C6	124.0 (2)	C7—C6—H6B	109.2
O1—C7—C6	111.19 (19)	H6A—C6—H6B	107.9
N4—N1—N2	110.82 (19)	H3A—O3—H3B	107 (3)
			
N1—N2—C5—N3	0.1 (3)	N1—N4—N3—C6	178.50 (19)
N3—N4—N1—N2	−0.3 (3)	C5—N3—C6—C7	−92.1 (3)
C5—N2—N1—N4	0.1 (3)	N4—N3—C6—C7	90.3 (3)
N2—C5—N3—N4	−0.3 (3)	O2—C7—C6—N3	−4.4 (3)
N2—C5—N3—C6	−178.2 (2)	O1—C7—C6—N3	176.12 (18)
N1—N4—N3—C5	0.3 (3)		

### Hydrogen-bond geometry (Å, º)

**Table:**

*D*—H···*A*	*D*—H	H···*A*	*D*···*A*	*D*—H···*A*
O3—H3*B*···N2	0.83 (4)	2.11 (4)	2.864 (3)	150 (3)
O1—H1*A*···O3^i^	0.89 (4)	1.72 (4)	2.603 (3)	175 (4)
O3—H3*A*···O2^ii^	0.76 (3)	2.00 (3)	2.752 (3)	168 (3)

Symmetry codes: (i) *x*, *y*, *z*+1; (ii) −*x*+1/2, *y*−1/2, *z*−1/2.
